# Regulation of Nucleolar Activity by MYC

**DOI:** 10.3390/cells11030574

**Published:** 2022-02-07

**Authors:** Isabella N. Brown, M. Carmen Lafita-Navarro, Maralice Conacci-Sorrell

**Affiliations:** 1 Department of Cell Biology, University of Texas Southwestern Medical Center, Dallas, TX 75390, USA; Isabella.Brown@UTSouthwestern.edu; 2 Hamon Center for Regenerative Science and Medicine, University of Texas Southwestern Medical Center, Dallas, TX 75390, USA; 3 Harold C. Simmons Comprehensive Cancer Center, University of Texas Southwestern Medical Center, Dallas, TX 75390, USA

**Keywords:** MYC, ribosome, ribosome biogenesis, nucleolus, translation, cell growth

## Abstract

The nucleolus harbors the machinery necessary to produce new ribosomes which are critical for protein synthesis. Nucleolar size, shape, and density are highly dynamic and can be adjusted to accommodate ribosome biogenesis according to the needs for protein synthesis. In cancer, cells undergo continuous proliferation; therefore, nucleolar activity is elevated due to their high demand for protein synthesis. The transcription factor and universal oncogene MYC promotes nucleolar activity by enhancing the transcription of ribosomal DNA (rDNA) and ribosomal proteins. This review summarizes the importance of nucleolar activity in mammalian cells, MYC’s role in nucleolar regulation in cancer, and discusses how a better understanding (and the potential inhibition) of aberrant nucleolar activity in cancer cells could lead to novel therapeutics.

## 1. Nucleolar Structure and Organization Allow for Flexibility in the Rate of Ribosome Biogenesis

The nucleolus, one of the largest membraneless organelles, is located in the nucleus and is the home of all of the steps necessary for ribosomal biogenesis including rDNA transcription, ribosomal RNA (rRNA) processing, and the assembly of ribosomes. The lack of a membrane allows the nucleolus to be very dynamic, as its size and composition can adjust to the demands for protein synthesis. For example, in cases of hyperproliferation, a higher rate of protein synthesis and ribosome biogenesis must be achieved to allow for cell growth, and the nucleolus increases in size and density to accommodate those needs [1,2].

Nucleolar morphology and function are tightly regulated during the cell cycle. Nucleoli are most active in G2, the cell cycle phase that precedes cell division and when protein synthesis capacity is the highest [3]. Conversely, during prophase, when cells are dividing, the nucleoli are disassembled and their contents are dispersed to eventually be inherited by daughter cells produced through mitosis [4]. Nucleoli are then re-formed in the daughter cells through the activity of nucleolar organizer regions (NORs), which are 50 Kb- to 6 Mb-long regions containing rDNA copies. In humans, NORs are located on chromosomes 13, 14, 15, 21, and 22 [5]. Strikingly, there are around 200 rDNA genes per human haploid genome, making rRNA the most abundant RNA in a cell [6,7].

**Figure 1:**
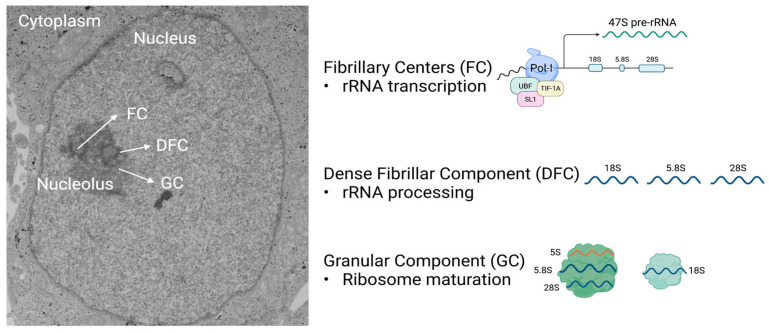
The nucleolus contains three distinct compartments responsible for pre-rRNA transcription, rRNA processing, and ribosome subunit assembly. The nucleoli are located within the nucleus (shown in the electron microscopy photo to the left) and are comprised of three sub-compartments: the fibrillary centers (FC), the dense fibrillar component (DFC), and the granular component (GC). The transcription of rDNA occurs in the FC upon the binding of selectivity factor 1 (SL1), which leads to the activation of the cofactors upstream binding factor (UBF) and TIF-1A, initiating RNAPolI to transcribe rDNA into 47S pre-rRNA. The 47S pre-rRNA is processed into 18S, 5.8S, and 28S rRNA in the DFC. Ribosome maturation proceeds in the GC, where additional RPs are wrapped around rRNAs. Figure created with Biorender.com (accessed on 31 January 2022).

Mammalian cells may contain one or more tripartite nucleoli, and each comprise three morphologically and biochemically distinct compartments that perform specific roles to produce ribosomal subunits: the fibrillary center (FC), the dense fibrillar component (DFC), and the granular component (GC). The FCs are encased in DFCs, which are surrounded by the GC (Figure 1). These three compartments work together to perform every step necessary to generate large (60S) and small (40S) ribosome subunits that comprise rRNA and ribosomal proteins (RPs) (Figure 1) [8]. Other organisms including yeast, invertebrates, fish, and reptiles have bipartite nucleoli, where the FCs are not present and rDNA genes are dispersed within the DFC [9,10,11,12,13]. The GC, which is the outermost layer of the nucleolus, is present in all species and is the site for the final steps in ribosome biogenesis. Through interactions with the nuclear pore complex, near-mature ribosome subunits are exported to the cytoplasm, where ribosome maturation is completed, thus allowing for the formation of complete ribosomes (80S) in the presence of mRNA [14,15,16]. Final ribosome maturation occurring in the cytoplasm prevents premature translation initiation until the ribosomes reach the cytoplasm.

The initial step of ribosome biogenesis begins in the FC when upstream binding factor (UBF) and selectivity factor 1 (SL1), also known as TIF-1B, and TIF-1A, bind to RNA polymerase I (RNAPolI) at the rDNA promoter. This initiates the transcription of the 47S pre-rRNA precursor, giving rise to 28S, 18S, and 5.8S mature rRNAs [17,18,19]. The synthesis of this 47S pre-rRNA as a single transcript allows for the stoichiometric production of the small and large ribosomal subunits, required at 1:1 ratio for translation. The 5S rRNA is the only rRNA not transcribed as part of 47S pre-rRNA; it is transcribed by the RNA polymerase III (RNAPolIII) in the nucleus [20,21].

The rRNAs undergo several steps of processing including methylations and pseudouridylations [22,23]. These modifications, which are guided by small nuclear RNAs (snoRNAs), are essential for the stabilization of secondary and tertiary rRNA structures, increasing translation accuracy [24,25,26]. Most steps in pre-rRNA maturation occur in the DFC where rRNA undergoes processing events that result in the mature rRNAs 28S and 5.8S, components of the large ribosome subunit, and 18S, a component of the small ribosome subunit [27]. The addition of RPs to rRNA begin co-transcriptionally. These RPs may be important in delimiting where the rRNA is cleaved. Other RPs are attached to the 28S and 18S in the GC of the nucleolus. All RP are transcribed from nuclear genes via the activity of the RNA polymerase II (RNAPolII). Once the RPs are translated in the cytoplasm, they are imported into the nucleus, where a subset of them bind to 5S rRNA. These are then co-imported into the GC of the nucleolus to be assembled into ribosomes. The mature 40S subunit comprises 32 small RPs, and the 60S subunit comprises 47 large RPs.

Errors in rDNA transcription, rRNA processing, and ribosome assembly are the cause of several disorders collectively named ribosomopathies. These include degenerative diseases such as abdominal aortic aneurysm [28,29] and Parkinson’s disease [30], along with developmental disorders such as Treacher Collins syndrome [31,32], Diamond-Blackfan anemia [33,34,35], Bowen-Conradi syndrome [36], and autosomal recessive primary microcephaly [37].

## 2. Nucleolar Size and Activity Are Increased in Hyperproliferative Cells

The presence of nutrients and growth factors in the cellular environment leads to the activation of signaling pathways that promote cell growth, including the production of new ribosomes. Hyperproliferative cancer cells often display activated growth factor signaling that can induce elevated RNAPolI–mediated rDNA transcription [38,39,40], leading to an increase in ribosome production. Moreover, alterations in the number of rDNA or 5S rRNA loci, documented in cancer cells, have the potential to increase ribosome production [41,42]. Elevated ribosome biogenesis causes morphological changes in the nucleoli that can be used for cancer diagnostics. Cancer cells have dark, dense, and large nucleoli, and these features are used by pathologists when grading solid tumors [2,14,43].

**Figure 2:**
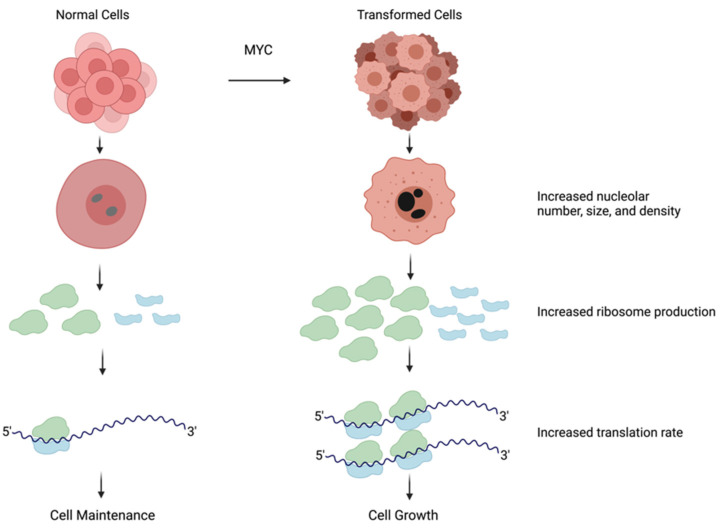
Transformed cells have larger nucleoli and increased ribosome production. The nucleolus increases in size and density to accommodate the need for ribosome production. In transformed cells frequently due to hyperactivation of MYC, nucleoli are larger in size and darker in color, indicating higher activity, which results in an increase in ribosome number. Containing a larger number of ribosomes amplifies mRNA translation and ultimately leads to an increase in cell growth. Figure created with Biorender.com (accessed on 5 January 2022).

Due to the high demand for ribosomes, transformed cells hyperactivate nearly all steps in ribosome biogenesis including rDNA transcription, rRNA processing, and the expression of RPs [1]. These changes are correlated with the activation of oncogenes and the inactivation of tumor suppressors. For example, the loss of tumor suppressors, such as p53 or PTEN, may lead to an increase in rDNA transcription, thus promoting ribosome biogenesis [44]. The oncogene MYC is one of the most potent drivers of ribosome biogenesis owing to its ability to concurrently promote the transcription of rDNA as well as genes encoding for ribosomal components and key regulators of ribosome biogenesis (Figure 2) [40,45,46,47].

## 3. The MYC Family of Transcription Factors Are Key Regulators of Cell Growth

MYC was originally discovered as a viral oncogene (*v-myc*) that caused myelocytomatosis, leukemia, and sarcoma [48]. Interestingly, *v-myc* was acquired from a cellular oncogene dubbed c-*myc*, now referred as MYC [49,50,51,52]. Subsequently, increasing evidence indicated that, in humans, MYC played a role in tumorigenesis without involving a viral infection [53]. Since then, there has been a tremendous effort to better understand MYC’s role under both normal and cancerous conditions. The discovery that cancer was not a contagious disease, but that viruses had the ability to capture and carry mammalian genes was groundbreaking. The attention then turned to developing an understanding of the mechanisms by which MYC promotes deregulated growth and cell transformation.

**Figure 3:**
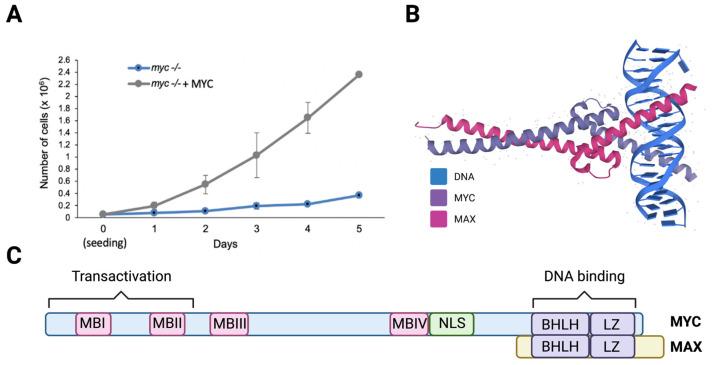
MYC heterodimerizes with MAX and increases cell proliferation. (**A**) Proliferation curve of HO15.19 *myc*-/- rat fibroblasts expressing empty vector or MYC. Cells were seeded and counted for 5 days. (**B**) The structure of the basic helix-loop-helix and leucine zipper (bHLH-LZ) domains of the heterodimer MYC-MAX and DNA (PDBe-KD), https://www.ebi.ac.uk/pdbe/pdbe-kb/proteins/P01106/interactions, (accessed on 28 November 2021). (**C**) Schematic representation of the MYC protein sequence with MYC Boxes (MBI, MBII, MBIII, MBIV), nuclear localization signal (NLS), and bHLHLZip domain on the C-terminus where DNA and MAX interacts. Figure created using Biorender.com (accessed on 28 January 2022).

MYC was found to be elevated in 70% of human cancers, making it one of the most pervasive oncogenes [54]. Supporting the correlation between MYC and cell growth, knocking out *myc* in cultured cells, including fibroblasts, causes a dramatic reduction in proliferation, and the reconstitution of MYC by ectopic expression rescues this phenotype (Figure 3A). Cells expressing elevated MYC levels are highly proliferative, and exhibit enlarged and active nucleoli [55].

The MYC family of proteins includes three transcription factors (MYC, MYC-N, and MYC-L) that regulate key biological processes; specifically, MYC and MYCN are essential for embryonic development [56,57]. Members of the MYC family contain a basic helix-loop-helix and leucine zipper (bHLHZip) domain in their C-terminal region that creates an alpha-helix structure through which they heterodimerize with MAX, a small bHLHZ protein [58,59,60,61] (Figure 3B). MYC-MAX heterodimers bind to and directly stimulate the transcription of genes that contain E-boxes (5′- CACGTG-3′) in their promoters [62,63]. There is evidence that MYC binds to non-E-box DNA sequences, with or without heterodimerizing MAX, via interacting with other DNA binding proteins such as RNAPolIII subunit TFIIIB [64,65,66]. MYC-NICK, a cytoplasmic proteolytic product of MYC that lacks the DNA binding domain and thus, is unable to regulate gene transcription, was also shown to play a pro-survival role in cancer cells [67,68,69].

All MYC variants contain highly conserved regions called MYC boxes (MB) (Figure 3C) that are important for transcription and protein stability [70]. MYC proteins have half-lives of around 30 min in normal cells and are considered unstable [71]. MBI contains a MYC degron, harboring the amino acids T58 and S62, which are phosphorylated preceding its targeting for ubiquitin-mediated degradation by its binding to FBWX7, a substrate adaptor protein for the SCF E3-ligase complex [72]. Mutations in MYC’s degron, or its E3 ligases, inhibits MYC degradation, causing its stabilization, which then contributes to cell transformation [73,74]. Moreover, the mutational inactivation of FBWX7 in tumors [75,76,77] causes the stabilization of MYC and other oncogenes such as Notch, cyclin E, and c-Jun that cooperate to drive cellular transformation [78,79,80,81,82,83].

MBII is a crucial region where histone acetyltransferase (HAT) complexes bind. HATs promote histone acetylation, which opens chromatin, facilitating access by the transcription machinery and ultimately activating gene transcription [84,85,86]. MYC increases local acetylation by binding to acetyltransferase complexes that acetylate histones H3 and H4 [87]. MYC specifically interacts with transcription-domain-associated-protein (TRRAP) [88], general control of amino acid synthesis protein 5 (GCN5) [66], Tat Interacting Protein, 60kDa (TIP60, also known as KAT5), and CREB-binding protein (p300/CBP) acetyltransferases [89]. MBIII and MBIV are less studied but are proposed to be important for the regulation of apoptosis and transcription by MYC [90,91].

## 4. MYC Promotes Transcription of rDNA

For proliferating cells to meet the ribosome and protein synthesis demand, rRNA production must be maintained at a high rate (Figure 2). The regulation of rDNA transcription by MYC has been documented in *Drosophila,* as well as in vertebrates [15]. MYC aids in rDNA transcription in two ways. First, MYC binds to rDNA loci together with RNAPolI, which facilitates transcriptional activation [46]. Second, MYC also enhances rDNA transcription by binding at the promoters of the RNAPolI cofactors SL1, UBF, and TIF-1A [39,56], which are often elevated in cancers, likely resulting from MYC transcriptional activity. UBF activates RNAPolI by stimulating transcriptional elongation [92] and SL1 stabilizes UBF and facilitates the pre-initiation complex formation on the rDNA promoter [93]. Therefore, elevated MYC in tumors can promote the activation of RNAPolI to transcribe rDNA into pre-rRNA (47S). Moreover, MYC facilitates the transcription of RNAPolIII–mediated 5S rRNA and transfer RNAs (tRNA), which carry amino acids to the translation machinery [65]. Additionally, MYC directly promotes the transcription of *TFIIIB*, critical for the activity of RNAPolIII [56]. While MYC induces transcription through RNA PolI, PolII, and PolIII activation, it also plays a role in stimulating rDNA transcription via chromatin remodeling mechanisms [94,95,96,97,98]. Through these multiple mechanisms, MYC can drive a dramatic increase in rRNA production, contributing to ribosome biogenesis in cancer cells.

## 5. MYC Promotes the Transcription of Genes Encoding RPs and Regulators of Nucleolar Assembly and Activity

**Figure 4:**
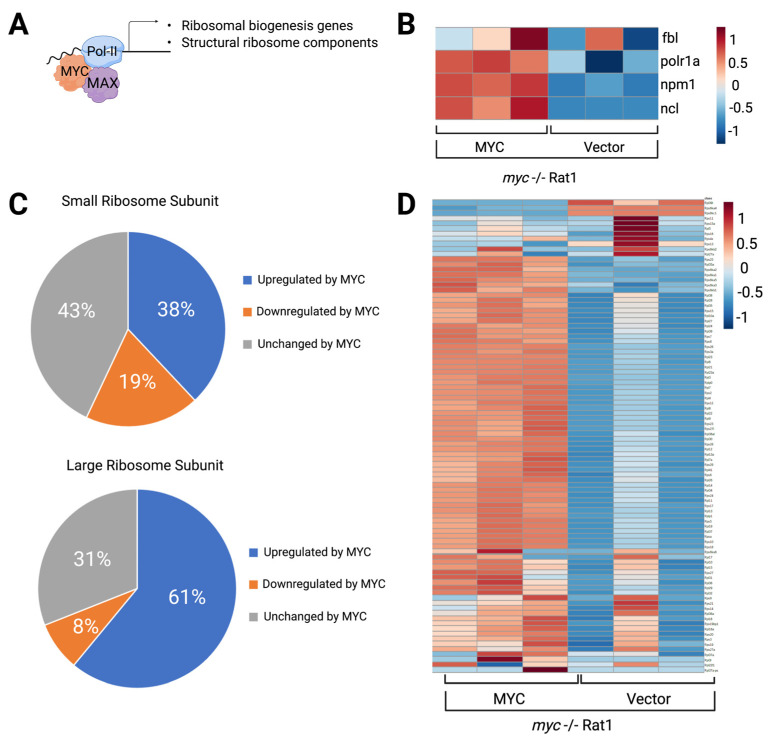
Ribosomal biogenesis and structural ribosome components are upregulated in MYC-expressing cells. (**A**). Schematic of the interaction of MYC and MAX with the transcription machinery that drives the expression of regulatory and structural genes necessary for ribosome biogenesis. (**B**). Heatmap of *myc*-/- expressing empty vector or reconstituted with MYC. Data are extracted from published RNAseq [55] for nucleolar genes. (**C**). Pie chart showing that MYC increased the transcription of 38% of the structural components of the small ribosome subunit and 61% of the large subunit. Data were obtained by comparing the expression of structural ribosome genes in *myc*-/- expressing empty vector or reconstituted with MYC using a cutoff of Log_2_ fold change of ≥or ≤0.58 and with adjusted *p*-value of ≤0.05. (**D**). Heatmap of *myc*-/- expressing empty vector or reconstituted by MYC. Data was extracted from published RNAseq [55] for structural RPs. Heatmaps were generated by MetaboAnalyst 5.0 (https://www.metaboanalyst.ca/, accessed on 10 December 2021). Figure created using Biorender.com, (accessed on 31 January 2022).

MYC controls the expression of about 20% of the genome, including genes that are crucial for nucleolar activity [15,55,99,100,101]. By comparing the transcriptional signature of *myc*-/- fibroblasts expressing empty vector or reconstituted with MYC, we found that MYC promotes the expression of the regulators of nucleolar activity (Figure 4A,B), as well as the structural components of the ribosome (Figure 4C,D). The expression of 38% of the genes encoding for small RPs and 61% of the genes encoding for large RPs are increased in MYC-expressing cells. It is likely that by increasing the expression of structural components of the ribosome, MYC supports an increase in ribosome subunit assembly and in protein synthesis. Moreover, MYC regulates the expression of numerous regulators of nucleolar assembly and activity [102]. For example, MYC promotes the transcription of *nucleolin* (*NCL*), which is needed for the processing of 47S pre-rRNA [103]. Furthermore, MYC directly regulates the expression of *nucleophosmin* (*NPM1*) and *fibrillarin* (*FBL*). Due to their biophysical properties, NPM1 and FBL separate into immiscible phases, contributing to the assembly of the GC and DFC, respectively (Figure 4B) [104,105]. The expression of these proteins must scale up to increase nucleolar size and function in cancer cells [2,43]. FBL and NPM1 were shown to be necessary for rRNA processing and the transport of 40S and 60S subunits into the cytoplasm [106,107]. Whether these functions of FBL and NPM1 are independent, or a result of their properties in assembling the nucleolar phases remains to be determined. This suggests that MYC, in addition to promoting the production of rRNA and RPs (Figure 4), may participate in regulating nucleolar assembly via the induction in FBL and NPM1.

## 6. MYC Promotes the Expression of Genes That Activate Ribosome Biogenesis and Protein Synthesis

In addition to directly regulating the transcription of rDNA and RPs, MYC promotes the transcription of additional transcription factors that regulate one or more aspects of nucleolar function. For example, MYC binds to the promoter of the transcription factor *aryl hydrocarbon receptor* (*AHR*) and of its heterodimeric partner *aryl hydrocarbon receptor nuclear translocator* (*ARNT*) and promotes their expression [55]. AHR in turn regulates the expression of genes involved in rDNA transcription such as *nucleolar and coiled-body phosphoprotein 1* (*NOLC1)*, rRNA processing such as *periodic tryptophan protein 2 homolog* (*PWP2)*, and protein synthesis such as *2-oxoglutarate and iron dependent oxygenase domain containing 1* (*OGFOD1)* in MYC-expressing cells [55]. Consequently, knocking down *AHR* in MYC-expressing cells causes decreased proliferation, nucleolar disassembly, and reduced protein synthesis. This suggests that MYC regulates nucleolar activity, and thus protein synthesis, partly through the transcriptional regulation of *AHR*. Moreover, AHR also regulates the production of nucleotides that are necessary for the elevated rate of rRNA production in cancer cells [108,109]. *Carbamoyl-phosphate synthetase 2, aspartate transcarbamylase,* and *dihydroorotase* (*CAD)*, *dihydroorotate dehydrogenase* (*DHODH),* and *uridine monophosphate synthetase* (*UMPS),* encoding for the enzymes of the *de novo* pyrimidine biosynthesis pathway [110], were previously found to be induced in MYC-expressing cells [101,111,112]. Recently, AHR was found to bind to the promoters of *DHODH* and *UMPS* and to cooperate with MYC to transcribe these genes. Knocking down *AHR* in MYC-expressing cells decreases the expression of *DHODH* and *UMPS* and the levels of the pyrimidine nucleotide uridine 5-monophosphate (UMP), which affects the number of pyrimidines needed for rRNA synthesis [101,108]. Therefore, AHR [55] activates the expression of *de novo* pyrimidine biosynthesis to partially support nucleolar activity. MYC was also found to regulate the transcription of genes encoding for factors responsible for translation elongation and initiation factors such as *eif4e* [102,113,114]. These factors are essential in translation and help carry out this process by binding to the cap structure at the 5′ end of mRNA, initiating the first step in translation [115].

## 7. Nucleolar Assembly and Function Regulate MYC Levels and Activity

**Figure 5:**
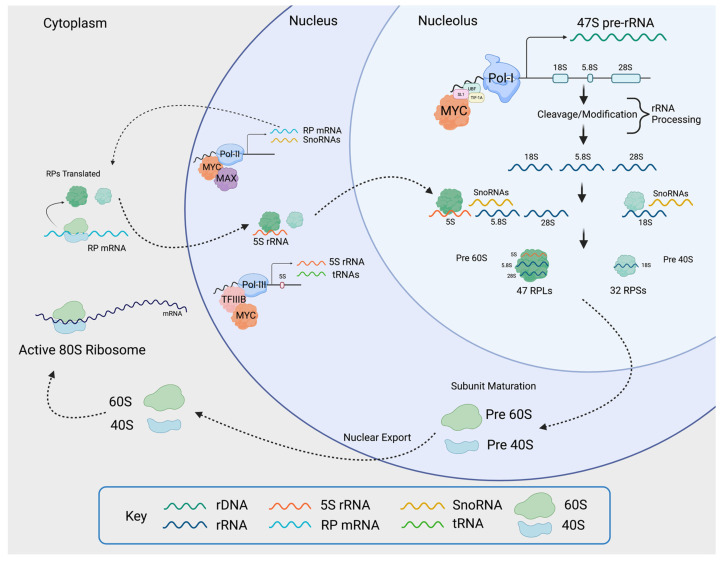
MYC induces ribosomal biogenesis processes. MYC heterodimerizes with MAX and promotes RNAPolI activity by binding to the rDNA promoter, as well as by activating the expression of *selectivity factor 1* (*SL1*), which binds other RNAPolI cofactors such as upstream binding factor (UBF) and TIF-1A. The rDNA is transcribed into 47S pre-rRNA in the nucleolus. The pre-rRNA is processed and cleaved into 18S, 5.8S, and 28S rRNA. MYC-MAX simultaneously enhances RNAPolII activity by binding to RNAPolII-regulated promoters, as well as RNAPolIII activity by inducing the expression of the RNAPolIII cofactor TFIIIB. This yields RNAPolII-driven small RPs (RPS) mRNA, large RPs (RPL) mRNA, RNAPolIII-driven snoRNAs, and 5S rRNA. The mRNAs are transported into the cytoplasm where mature ribosomes translate them into small and large RPs. Once translated, small RPs are imported into the nucleolus. Large RPs are first imported into the nucleus, where they interact with 5S rRNA, and then to the nucleolus. In the nucleolus, the pre-rRNAs are modified and processed with the help of snoRNAs. As they are maturing, the rRNA wrap around the RPs, creating the pre-40 and pre-60 subunits which are exported to the cytoplasm for the final maturation step. Once maturation is complete in the cytoplasm, the active 80S ribosomes are formed. The small 40S subunit comprises 18S rRNA and 32 small RPs, and the large 60S subunit comprises 5S, 5.8S, and 28S rRNA and 47 large RPs. Figure created using Biorender.com (accessed on 31 January 2022).

As discussed above, MYC was shown to regulate multiple aspects of nucleolar function, including transcription of rDNA and RPs genes (Figure 5). Conversely, nucleolar components are also shown to affect MYC levels and activity, thus creating a tight positive feedback loop: MYC promotes nucleolar activity, and nucleolar components regulate MYC levels and function. For example, MYC induces the expression of *NPM1* [116] and NPM1 in turn regulates MYC activity and stability through directly interacting with MYC, which stimulates its binding to target gene promoters such as *eif4e*, *ncl*, and *cdk4* [107,116,117]. Furthermore, NPM1 was proposed to be necessary for the ability of MYC to induce rRNA synthesis in the nucleolus. Thus, constitutive NPM1 overexpression stimulates MYC-mediated rRNA synthesis [117]. Conversely, other studies have shown that the elevated expression of NPM1 enhances the nucleolar localization of MYC, and that this is necessary for the FBXW7γ-mediated degradation of MYC in the nucleolus [72,117]. FBXW7γ was reported to be localized in the nucleolus where it colocalizes with the nucleolar pool of MYC. The loss of FBXW7γ in cancer cells leads to MYC stabilization which likely enhances rRNA production [72].

Additionally, a feedback loop between MYC and ribosomal proteins such as RPL5 and RPL11 was also documented. MYC activity was found to be altered by RPL11, which is capable of binding to MYC, thereby inhibiting its transcriptional activity [118]. Moreover, RPL5 was shown to facilitate the degradation of MYC mRNA by linking its 3′UTR to the RISC RNA degradation complex [119]. Further studies are needed to generate a more comprehensive view of how nucleolar resident proteins and ribosomal factors control the activity of MYC and of other growth-promoting factors in normal and cancer cells.

## 8. Targeting Aberrant Nucleolar Activity to Inhibit Cancer Growth

As a result of the extensive effects of MYC on ribosome biogenesis, MYC-expressing cells generally have larger and hyperactive nucleoli [43]. Therefore, MYC-dependent tumors are likely sensitive to the inhibition of nucleolar activity, making the inhibition of excessive ribosome biogenesis in tumors an attractive approach for cancer therapeutics. Nevertheless, inhibiting nucleolar activity could lead to undesirable effects given the requirement for ribosomes in normal cells. Hence, identifying and inhibiting tumor-specific nucleolar regulators (possibly driven by MYC) may lead to the development of novel strategies to block cancer cell growth.

Interestingly, some drugs, including cisplatin, oxaliplatin, and doxorubicin, were shown to have off-target effects that inhibit RNAPolI activity, altering rRNA production [120,121,122,123]. The topoisomerase II inhibitor ellipticine was shown to impair SL1 promoter binding, halting RNAPolI–mediated transcription, thus causing dramatic effects on ribosome biogenesis in vitro and in pre-clinical models [56]. Nevertheless, phase I and II clinical trials revealed unacceptable cytotoxicity; therefore, this is not a safe therapeutic option [123,124]. Currently, there is a focus on identifying drugs that target the nucleolus of cancer cells in a more specific manner, initiating a promising option for the treatment of human tumors [123,125,126,127,128,129].

Interfering with rRNA and ribosome production can alter the homeostasis and integrity of the nucleolus, activating nucleolar stress that causes cell cycle arrest and apoptosis. The best-known mechanism of nucleolar stress is mediated by p53 [130]. Under normal cellular conditions, the E3 ubiquitin ligase mouse double minute 2 (MDM2) interacts with p53, targeting it for degradation [130]. In contrast, upon nucleolar stress, RPL5, RPL11, and the 5S rRNAs redistribute from the nucleolus to the nucleoplasm where they sequester MDM2. This prevents MDM2-p53 interaction, leading to p53 stabilization, which in turn causes cell cycle arrest and apoptosis [131,132]. In line with this idea, an emerging approach to cause nucleolar stress in cancer cells is to block *de novo* nucleotide biosynthesis, which limits nucleotide availability and thus impairs rRNA production affecting nucleolar activity. For instance, work with small-cell lung cancer (SCLC) models showed that SCLC with elevated MYC expression were more sensitive to the inhibition of the *de novo* purine biosynthesis enzyme *inosine-5′-monophosphate dehydrogenase* (IMPDH1/2), which affected rRNA production and thus, cell proliferation [133,134]. Inhibition of the *de novo* pyrimidine biosynthesis enzyme DHODH has been shown to decrease the production of rRNA, to induce nucleolar disassembly, and to stabilize p53 in glioblastoma, breast, and colon cancer cells, leading to a decrease in proliferation and an increase in apoptosis [108,109]. Additional pharmacologic inhibitors may be exploited to limit nucleolar activity in cancer cells. For example, MLN4924, a chemical that inhibits neddylation, a post-translational modification that regulates the activity of cullins (scaffold components for RING E3-ubiquitin ligases), has been shown to increase p53 levels by altering the MDM2-RPL11 pathway [135]. During phase 1 clinical trials, it was found that this drug held promise as a useful cancer therapeutic, although further clinical trial phases need to be conducted [135,136]. Whether MLN4924 causes nucleolar stress in patients is yet to be determined. Altogether, targeting nucleolar activity in tumors with the aim of inducing nucleolar stress may be a potent strategy to inhibit the growth of tumors that depend on MYC, and potentially, other oncogenes as well.

## 9. Future Directions

In recent years, the concept of ribosome heterogeneity has emerged [137], and understanding its potential role in cancer could lead to new therapeutic strategies. When ribosomes were first discovered, it was suggested that there was a universal ribosome for every protein formed [138], and the concept that ribosomes have no specificity or regulatory functions was widely accepted [137,139]. However, recent groundbreaking work has shown that ribosome heterogeneity might play an important regulatory role in cells after all [140,141,142]. There is supportive evidence that ribosomes are not always composed of the same RPs; however, the details are not fully mapped [137,143,144,145]. Additionally, several RPs have known variants and pseudogenes that may perform specific functions [146]. Some of these RP variants and pseudogenes are expressed in specific tissues, which could potentially contribute to ribosome heterogeneity [145,146]. With this concept in mind, it has also been noted that some RPs have extra-ribosomal functions including DNA repair, development regulation, cell growth and apoptosis regulation, tumor suppressor gene and proto-oncogene regulation, and RNA splicing and modification [147,148]. Many tumor types have been characterized with an increase in RPs and specific RPs mutations [149,150]. In about 10% of T-cell acute lymphoblastic leukemia cases, patients have a frameshift mutation in RPL22, which is thought to contribute to cancer progression [151]. In colorectal cancer, the elevation of some specific small RPs, including RPS3, RPS6, RPS8, and RPS12, increases ribosome biogenesis and possibly leads to the activation of extra-ribosomal functions such as DNA replication, RNA splicing and modification, and cell growth [148,152].

The concept that ribosomes can be specialized may lead to a new research avenue for the development of drugs that target cancer-specific ribosomes. Whether MYC or other oncogenes regulate the production of tumor-specific ribosomes is yet to be determined. If such ribosomes exist, this will provide a completely new area of investigation with potential for novel strategies to target tumor cells.

## Data Availability

Not applicable.
